# Spatial processing in binocular rivalry

**DOI:** 10.1186/1471-2202-13-S1-P166

**Published:** 2012-07-16

**Authors:** Phyllis M Thangaraj, Shashaank Vattikuti, Carson C Chow

**Affiliations:** 1 Laboratory of Biological Modeling, National Institute of Diabetes and Digestive and Kidney Diseases, National Institutes of Health, Bethesda, MD 20892, USA

## 

In the presence of ambiguous visual stimuli, cortical neurons will exhibit rivalry, normalization or winner-take-all dynamics. Binocular rivalry describes the oscillation of image perception that occurs when a different image is presented to each eye simultaneously [1]. However, rivalry only occurs if the two images are spatially co-localized. If the images are spatially separated then the neural activity will exhibit normalization, where the activity of a neuron is a sub-linear sum of the activities when each image is presented separately. Population rate models can reproduce the these three phases [2] and phase transitions occur under changes in the mutual inhibition strength between neurons.

Measured neuronal activity that is most strongly correlated with the observed percept is generally located in higher visual areas such as inferotemporal cortex. Hence, the mechanism that causes neurons to exhibit rivalry when images are spatially co-localized and normalization when they are not is not understood. Until recently, the recognition of an object by the inferotemporal cortex was considered to be an independent process from determining the object’s position in space. This assumption corresponded with the observation that object recognition does not change significantly when the object moves within the visual field. Evidence had been mounting against this assumption, beginning with the finding that IT neurons have receptive fields and are sensitive to changes in position [3]. Through neuroimaging of the cortex and behavioral testing, it was determined that object representation in the inferotemporal cortex does in fact depend on position [4].

This study creates a three dimensional model of binocular rivalry in which the neurons respond to specific two dimensional positions in space and a particular stimulus type. Past models of rivalry have included two-dimensional positions of neurons [5]. This model describes how the position of rivaling images can influence the strength and timing of binocular rivalry. By encoding spatial preferences into the neurons, this model is able to simulate binocular rivalry initiation through changes in object position in addition to changes in inhibition strength. Based on an image’s position, certain neurons may not be directly stimulated, but may spike anyway due to their connections with stimulated neurons, causing a transition into binocular rivalry from normalization. By encoding spatial preferences into the neurons, this model allows phase transitions to occur through changes in object position in addition to changes in inhibition strength.
